# *SOX9 *gene transfer via safe, stable, replication-defective recombinant adeno-associated virus vectors as a novel, powerful tool to enhance the chondrogenic potential of human mesenchymal stem cells

**DOI:** 10.1186/scrt113

**Published:** 2012-06-28

**Authors:** Jagadeesh K Venkatesan, Myriam Ekici, Henning Madry, Gertrud Schmitt, Dieter Kohn, Magali Cucchiarini

**Affiliations:** 1 Center of Experimental Orthopaedics, Saarland University Medical Center, Kirrbergerstr. Bldg 37, Homburg/Saar, D-66421 Germany; 2 Department of Orthopaedic Surgery, Saarland University Medical Center, Kirrbergerstr. Bldg 37, Homburg/Saar, D-66421 Germany

## Abstract

**Introduction:**

Transplantation of genetically modified human bone marrow-derived mesenchymal stem cells (hMSCs) with an accurate potential for chondrogenic differentiation may be a powerful means to enhance the healing of articular cartilage lesions in patients. Here, we evaluated the benefits of delivering *SOX9 *(a key regulator of chondrocyte differentiation and cartilage formation) via safe, maintained, replication-defective recombinant adeno-associated virus (rAAV) vector on the capability of hMSCs to commit to an adequate chondrocyte phenotype compared with other mesenchymal lineages.

**Methods:**

The rAAV-FLAG-h*SOX9 *vector was provided to both undifferentiated and lineage-induced MSCs freshly isolated from patients to determine the effects of the candidate construct on the viability, biosynthetic activities, and ability of the cells to enter chondrogenic, osteogenic, and adipogenic differentiation programs compared with control treatments (rAAV-*lacZ *or absence of vector administration).

**Results:**

Marked, prolonged expression of the transcription factor was noted in undifferentiated and chondrogenically differentiated cells transduced with rAAV-FLAG-h*SOX9*, leading to increased synthesis of major extracellular matrix components compared with control treatments, but without effect on proliferative activities. Chondrogenic differentiation (*SOX9*, type II collagen, proteoglycan expression) was successfully achieved in all types of cells but strongly enhanced when the *SOX9 *vector was provided. Remarkably, rAAV-FLAG-h*SOX9 *delivery reduced the levels of markers of hypertrophy, terminal and osteogenic/adipogenic differentiation in hMSCs (type I and type X collagen, alkaline phosphatise (ALP), matrix metalloproteinase 13 (MMP13), and osteopontin (OP) with diminished expression of the osteoblast-related transcription factor runt-related transcription factor 2 (RUNX2); lipoprotein lipase (LPL), peroxisome proliferator-activated receptor gamma 2 (PPARG2)), as well as their ability to undergo proper osteo-/adipogenic differentiation. These effects were accompanied with decreased levels of β-catenin (a mediator of the Wnt signaling pathway for osteoblast lineage differentiation) and enhanced parathyroid hormone-related protein (PTHrP) expression (an inhibitor of hypertrophic maturation, calcification, and bone formation) via *SOX9 *treatment.

**Conclusions:**

This study shows the potential benefits of rAAV-mediated *SOX9 *gene transfer to propagate hMSCs with an advantageous chondrocyte differentiation potential for future, indirect therapeutic approaches that aim at restoring articular cartilage defects in the human population.

## Introduction

Adult hyaline articular cartilage that allows smooth gliding and weight-bearing on articulating surfaces is an aneural and avascular tissue, lacking a lymphatic drainage. As a consequence, articular cartilage does not have access to reparative cells brought in other tissues in response to injury, and articular cartilage defects become persistent and progress over time after trauma or degeneration. The chondrocytes are the only cells present in the articular cartilage, producing and surrounding themselves with an intricate network of extracellular matrix composed mostly of proteoglycans and type II collagen that is largely deteriorated in cartilage lesions. Despite several currently available surgical options, restoration of a native structure and phenotype in injured articular cartilage is difficult to achieve, as only a poorly organized repair tissue made of type I collagen is produced, which does not totally integrate with the surrounding cartilage and does not withstand mechanical stress over time.

The principle of transplanting progenitor cells like mesenchymal stem cells (MSCs) to improve the regenerative properties of the articular cartilage is an attractive approach to enhance the natural healing response of damaged tissue [1]. MSCs have a strong potential for self-renewal and differentiation into various cell lineages, among which are the chondrocytes. They can be easily isolated and propagated, may recapitulate lineage transitions originally involved in tissue formation, and might be better suited than differentiated cells, such as chondrocytes, that tend to lose their phenotype on expansion. Although MSCs have been safely applied in patients to treat articular cartilage defects and osteoarthritis [2,3] without signs of tumorigenicity or immunologic reactions, their use is still impeded by the low percentage of cells that undergo functional differentiation programs to produce adequate reparative tissues. Specifically, for articular cartilage repair, the important challenge when implanting MSCs will be to maintain the MSC-derived cells in a non-hypertrophic state that avoids premature terminal differentiation, hypertrophy, and ossification [4,5].

In this regard, gene-transfer methods might provide powerful tools to overcome such limitations by precisely and durably improving the intrinsic chondrogenic potential of MSCs for strategies that aim at enhancing articular cartilage repair. Different factors have been reported for their ability to direct MSCs toward the chondrocyte phenotype. They include the transforming growth factor beta (TGF-β) [6-11], bone morphogenic proteins (BMPs) [9,10,12,13], the insulin-like growth factor I (IGF-I) [14,15], basic fibroblast growth factor (FGF-2) [16,17], zinc-finger protein 145 (ZNF145) [18], human telomerase (hTERT) [19,20], and the antiapoptotic factor Bcl-xL [21]. Yet, the use of these agents remains disputable, as most of them do not promote the synthesis of specific cartilage matrix components *per se *(FGF-2, hTERT, Bcl-xL) [16,19-21] or an adequate chondrogenic differentiation (IGF-I) [15], or even lead to undesirable MSC hypertrophy (TGF-β, BMPs) [7,10,13,15]. In this regard, members of the sex-determining region Y-type high-mobility group box (SOX) family of transcription factors might be better candidates to refine chondrogenesis in MSCs. Especially SOX9 plays central roles in chondrocyte differentiation and cartilage formation [22] and has been reported for its inhibitory or delaying effects on terminal differentiation and hypertrophy [23-27], although recent evidence demonstrated that the impact of SOX9 might be different for postdifferentiation chondrocytes [28,29]. Interestingly, systems used to deliver *SOX *sequences to MSCs so far have been based on the use of nonviral [26,30] and classic virus-derived constructs, including adenoviral [24,31,32] and retro-/lentiviral vectors [33]. They display low (nonviral and retroviral vectors) and transient (nonviral and adenoviral vectors) gene-transfer efficiencies, tend to induce host immune responses (adenoviral vectors), or promote insertional mutagenesis (retroviral vectors). Vectors such as those generated from the nonpathogenic, replication-defective adenoassociated virus (AAV), instead, have considerable advantages for gene-transfer protocols in MSCs. Recombinant AAV (rAAV) has been shown to transduce these cells at very high efficiencies and over sufficient periods without impairing their differentiation potential [8,16,34]. They can be safely used because of the removal of potentially immunogenic viral sequences in the recombinant genome and may avoid the risk for insertional mutagenesis by being kept in stable episomal forms in the host, making rAAV an adapted system for experimental settings *in vivo *[8,35] and for clinical protocols [36].

In the present study, we examined the efficacy of rAAV to deliver an *SOX9 *gene sequence, a key regulator of chondrogenesis, to primary adult human MSCs isolated from patients, the ultimate targets for clinical applications. We also determined the effects of the candidate treatment on the proliferative, metabolic, and differentiative (chondrogenic versus osteo-/adipogenic) activities of these cells as a prelude for future evaluations *in vivo *to enhance articular cartilage repair by implanting such genetically modified progenitor cells in cartilage defects.

## Materials and methods

### Reagents

Reagents were from Sigma (Munich, Germany) unless otherwise indicated. Recombinant FGF-2 (rFGF-2) and TGF-β were purchased at R&D Systems (Wiesbaden-Nordenstadt, Germany). The dimethylmethylene blue dye was from Serva (Heidelberg, Germany). The anti-type I (AF-5610) and anti-type II collagen (AF-5710) antibodies were from Acris (Hiddenhausen, Germany), the anti-FLAG (BioM2) and anti-type X collagen (COL-10) antibodies from Sigma, and the anti-SOX9 (C-20), anti-CD34 (C-18), anti-CD71 (C-20), and anti-CD105 (T-20) antibodies from Santa Cruz Biotechnology (Heidelberg, Germany). Biotinylated secondary antibodies and ABC reagent were from Vector Laboratories (Alexis Deutschland GmbH, Grünberg, Germany). The Cell Proliferation reagent WST-1 was from Roche Applied Science (Mannheim, Germany). The type II and type I collagen enzyme-linked immunosorbent assays (ELISAs; Arthrogen-CIA Capture ELISA kit) were from Chondrex (Redmond, WA, USA), and the type X collagen ELISA (COL-10) from Antibodies-online GmbH (Aachen, Germany). The alkaline phosphatase (ALP) activity detection assay (QuantiChrom ALP Kit) was from BioAssay Systems (Biotrend Chemikalien GmbH, Cologne, Germany).

### Cell culture

Bone marrow aspirates (~15 ml) were obtained from the distal femurs of patients undergoing total knee arthroplasty (*n *= 28). The study was approved by the Ethics Committee of the Saarland Physicians Council. All patients provided informed consent before inclusion in the study. All procedures were in accordance with the Helsinki Declaration. Mesenchymal stem cells (MSCs) were isolated and expanded in culture by using standard protocols [16,37,38]. Aspirates were washed in DMEM, and the cell-containing fractions layered onto Histopaque-1077 density gradient and centrifuged at 800 *g *for 30 minutes at room temperature. The nucleated cell fraction at the interface was collected, washed, and resuspended in Mesencult Basal Medium containing MSC Stimulatory Supplements (StemCell Technologies, Cologne, Germany) with 100 U/ml penicillin and 100 μl/ml streptomycin (pen-strep) (basal medium) and rFGF-2 (10 ng/ml). MSCs were plated at 2 × 10^5 ^cells/cm^2 ^in T-75 flasks and maintained at 37°C in a humidified atmosphere with 5% CO_2_. The medium was exchanged after 48 hours and every 2 to 3 days thereafter. Cells were detached and replated for further experiments at appropriate densities. MSCs were analyzed with flow cytometry for expression of stem-cell surface markers (CD71^+^, CD105^+^, CD34^-^). All experiments were performed with cells at not more than passage two.

### Plasmids and rAAV vectors

The constructs were all derived from the same parental AAV-2 genomic clone, pSSV9 [39,40]. rAAV-*lacZ *is an AAV-2-based vector plasmid carrying the *lacZ *gene encoding β-galactosidase (β-Gal) under the control of the cytomegalovirus immediate-early (CMV-IE) promoter [16,35,41-44]. rAAV-FLAG-h*SOX9 *is the same AAV-2-based vector plasmid used to prepare rAAV-*lacZ *but carrying a FLAG-tagged *SOX9 *sequence (1.7 kb) [45] instead of *lacZ *[42,43]. All rAAVs were packaged as conventional (not self-complementary) vectors in the 293 cell line, an adenovirus-transformed human embryonic kidney cell line, by using Adenovirus 5 to provide helper functions in combination with the *trans*-acting AAV-2 factors for replication and encapsidation functions supplied by the pAd8 helper plasmid [40]. Potential contamination from Adenovirus was prevented by heating and purification, as previously described [16,35,41-44]. The preparations were dialyzed and titered with real-time PCR [16,35,41-44], averaging 10^11 ^functional units/ml (that is, 10^12 ^viral genomes/ml).

### rAAV-mediated gene transfer

Monolayer cultures of undifferentiated hMSCs (4 × 10^4 ^cells) were transduced with rAAV (40 μl vector) or left untreated [16,35,41-44] and kept in basal medium for up to 21 days. hMSC aggregate cultures (2 × 10^5 ^cells) were prepared [16,37,38] and kept in DMEM high glucose (4.5 g/L), pen-strep, ITS^+ ^Premix (insulin, 6.25 μg/ml; transferring, 6.25 μg/ml; selenous acid, 6.25 μg/ml; linoleic acid, 5.35 μg/ml; bovine serum albumin, 1.25 μg/ml), pyruvate (1 m*M*), ascorbate 2-phosphate (37.5 μg/ml), dexamethasone (10^-7 ^*M*), and TGF-β (10 ng/ml) (defined medium) at 37°C in a humidified atmosphere with 5% CO_2_. The cells formed a free-floating mass within 24 hours that was transduced with rAAV (100 μl vector) 1 day after aggregate formation (or left untreated) and kept in defined medium for up to 21 days [16]. For osteogenic and adipogenic differentiation, hMSCs in monolayer cultures (10^5 ^cells) were transduced with rAAV (100 μl vector) or left untreated, and induced toward osteogenic differentiation by using the StemPro Osteogenesis Differentiation kit (Life Technologies GmbH, Darmstadt, Germany) or adipogenic differentiation by using the StemPro Adipogenesis Differentiation kit (Life Technologies GmbH) for up to 21 days at 37°C in a humidified atmosphere with 5% CO_2 _[38,46,47].

### Histology, immunocyto-, and immunohistochemistry

Monolayer and aggregate cultures were harvested and fixed in 10% buffered formalin. Aggregates were dehydrated in graded alcohols, embedded in paraffin, and sectioned (5 μm). Samples were processed for transgene expression by immunocytochemical and immunohistochemical analyses using specific antibodies. Sections were also stained with H&E (cellularity), toluidine blue (matrix proteoglycans), and alizarin red (matrix mineralization) according to routine protocols. Expression of type I, type II, and type X collagen was detected by immunohistochemistry by using specific antibodies, biotinylated secondary antibodies, and the ABC method with diaminobenzidine (DAB) as the chromogen [16,37,38]. To control for secondary immunoglobulins, samples were processed with omission of the primary antibody. Osteogenically differentiated cultures were stained for ALP (Alkaline Phosphatase staining kit, Sigma), and adipogenically differentiated cultures for intracellular lipid droplets with Oil Red O (Sigma) [38,46,47]. Samples were examined with light microscopy (Olympus BX 45, Hamburg, Germany).

### Histomorphometry

The intensities of SOX9 immunostaining and the percentages of areas stained for ALP or Oil Red O were calculated as being the ratios of positively stained surface to the total surface evaluated. The cell numbers and viability were monitored with trypan blue exclusion and by counting cells on H&E-stained sections from aggregates [16,35,41-44]. The intensities of SOX9 immunostaining, transduction efficiencies (X-Gal staining), aggregate diameters, cell densities, intensities of toluidine blue and of alizarin red staining, and those of type I, type II, and type X collagen immunostaining were measured at three standardized sites or by using 10 serial histologic and immunohistochemical sections for each parameter, test, and replicate condition by using SIS AnalySIS (Olympus), Adobe Photoshop (Adobe Systems, Unterschleissheim, Germany), and Scion Image (Scion Corporation, Frederick, MD, USA) [16,35,41-44]. The toluidine blue staining intensities were in pixels per area, and those for alizarin red staining, type I, type II, and type X collagen immunostaining in percentages, representing the ratios of positively stained tissue surface to the total surface of the site evaluated.

### Biochemical assays

Cultures were harvested with selective papain digestion for aggregates. Cell proliferation was assessed with the Cell Proliferation reagent WST-1 [16,44]. The DNA contents were determined with a fluorimetric assay by using Hoechst 33258 [16,35,41-44]. The proteoglycan contents were measured by binding to dimethylmethylene blue dye [16,35,41-44], and those for type I, type II, and type X collagen with ELISA [16,41-44]. The ALP activities were analyzed with a colorimetric assay to measure the hydrolysis of *p*-nitrophenol by using a standard curve made of this reagent [16]. All measurements were performed by using a GENios spectrophotometer/fluorometer (Tecan, Crailsheim, Germany).

### Total RNA extraction and real-time RT-PCR analyses

Total cellular RNA was extracted from the cultures by using the RNeasy Protect Mini Kit with an on-column RNase-free DNase treatment (Qiagen, Hilden, Germany) [16,37,46]. RNA was eluted in 30 μl RNase-free water. Reverse transcription was carried out with 8 μl of eluate by using the 1^st ^Strand cDNA Synthesis kit for RT-PCR (AMV) (Roche Applied Science). An aliquot of the cDNA product (2 μl) was amplified with real-time PCR by using the Brilliant SYBR Green QPCR Master Mix (Stratagene, Agilent Technologies, Waldbronn, Germany) [16] on an Mx3000P QPCR operator system (Stratagene) as follows: (95°C, 10 minutes), amplification by 40 cycles (denaturation at 95°C, 30 seconds; annealing at 55°C, 1 minute; extension at 72°C, 30 seconds), denaturation (95°C, 1 minute), and final incubation (55°C, 30 seconds). The primers (Invitrogen GmbH) used were SOX9 (chondrogenic marker) (forward 5'-ACACACAGCTCACTCGACCTTG-3'; reverse 5'-GGGAATTCTGGTTGGTCCTCT-3'), type II collagen (COL2A1) (chondrogenic marker) (forward 5'-GGACTTTTCTCCCCTCTCT-3'; reverse 5'-GACCCGAAGGTCTTACAGGA-3'), type I collagen (COL1A1) (osteogenic marker) (forward 5'-ACGTCCTGGTGAAGTTGGTC-3'; reverse 5'-ACCAGGGAAGCCTCTCTCTC-3'), type X collagen (COL10A1) (marker of hypertrophy) (forward 5'-CCCTCTTGTTAGTGCCAACC-3'; reverse 5'-AGATTCCAGTCCTTGGGTCA-3'), alkaline phosphatase (ALP) (osteogenic marker) (forward 5'-TGGAGCTTCAGAAGCTCAACACCA-3'; reverse 5'-ATCTCGTTGTCTGAGTACCAGTCC-3'), matrix metalloproteinase 13 (MMP13) (marker of terminal differentiation) (forward 5'-AATTTTCACTTTTGGCAATGA-3'; reverse 5'-CAAATAATTTATGAAAAAGGGATGC-3'), osteopontin (OP) (osteogenic marker) (forward 5'-ACGCCGACCAAGGAAAACTC-3'; reverse 5'-GTCCATAAACCACACTATCACCTCG-3'), runt-related transcription factor 2 (RUNX2) (osteogenic marker) (forward 5'-GCAGTTCCCAAGCATTTCAT-3'; reverse 5'-CACTCTGGCTTTGGGAAGAG-3'), β-catenin (mediator of the Wnt signaling pathway for osteoblast lineage differentiation) (forward 5'-CAAGTGGGTGGTATAGAGG-3'; reverse 5'-GCGGGACAAAGGGCAAGA-3'), parathyroid hormone-related protein (PTHrP) (hypertrophy-associated gene) (forward 5'-CGACGACACACGCACTTGAAAC-3'; reverse 5'-CGACGCTCCACTGCTGAACC-3'), lipoprotein lipase (LPL) (adipogenic marker) (forward 5'-GAGATTTCTCTGTATGGCACC-3'; reverse 5'-CTGCAAATGAGACACTTTCTC-3'), peroxisome proliferator-activated receptor gamma 2 (PPARG2) (adipogenic marker) (forward 5'-GCTGTTATGGGTGAAACTCTG-3'; reverse 5'-ATAAGGTGGAGATGCAGGCTC-3'), and glyceraldehyde-3-phosphate dehydrogenase (GAPDH) (housekeeping gene and internal control) (forward 5'-GAAGGTGAAGGTCGGAGTC-3'; reverse 5'-GAAGATGGTGATGGGATTTC-3') (all 150 n*M *final concentration) [13,15,16,46-49]. Control conditions included reactions using water and non-reverse-transcribed mRNA. Specificity of the products was confirmed by melting curve analysis and agarose gel electrophoresis. The threshold cycle (Ct) value for each gene of interest was measured for each amplified sample by using the MxPro QPCR software (Stratagene), and values were normalized to GAPDH expression by using the 2^-ΔΔCt ^method, as previously described [16].

### Statistical analysis

Data are expressed as mean ± standard deviation (SD) of separate experiments. Each treatment condition was performed in triplicate in three independent experiments for each patient. Data were obtained by two individuals that were blinded with respect to the treatment groups. The *t *test and the Mann-Whitney Rank Sum Test were used where appropriate. Any *P *value of less than 0.05 was considered statistically significant.

## Results

### Efficient and sustained *SOX9 *overexpression in monolayer cultures of undifferentiated human mesenchymal stem cells via rAAV-mediated gene transfer

**Figure 1:**
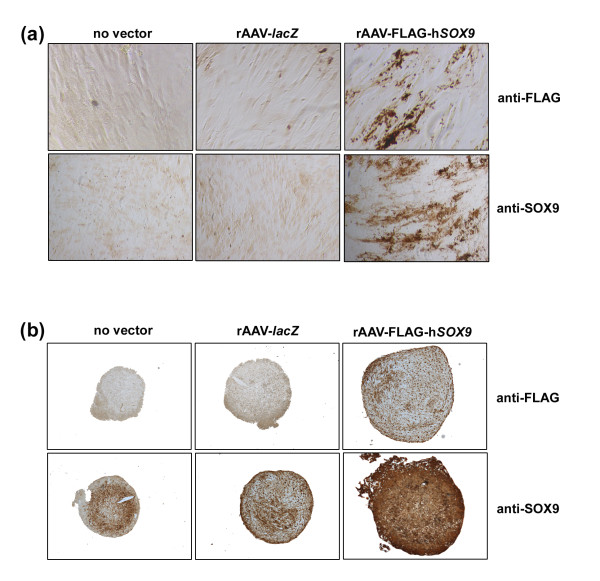
**Detection of rAAV-mediated transgene expression in undifferentiated monolayer and chondrogenically differentiated aggregate cultures of human mesenchymal stem cells (hMSCs)**. Cells were transduced with rAAV-*lacZ *or rAAV-FLAG-h*SOX9 ***(a) **in monolayer culture (40 μl each vector) or **(b) **in aggregate cultures (100 μl each vector), as described in Materials and methods, or left untreated, and processed to monitor transgene expression 21 days after vector application by analyzing the immunoreactivity to the FLAG tag or to SOX9. (**a**) Anti-FLAG at magnification ×20 and anti-SOX9 at magnification ×4; (**b**) magnification ×4.

Human adult mesenchymal stem cells (hMSCs) were first transduced with the candidate rAAV-FLAG-h*SOX9 *vector in undifferentiated monolayer cultures compared with control treatments (reporter rAAV-*lacZ *gene vector application or a condition lacking vector administration) to examine the ability of rAAV to mediate overexpression of the transcription factor over time in these cells *in vitro *at an undifferentiated stage. Sustained, intense immunoreactivity to the FLAG tag and SOX9 was detected only in cells transduced with rAAV-FLAG-h*SOX9 *compared with control applications [24] after 5 days (not shown) and for up to 21 days (Figure 1a). Transduction efficiencies ranged between 70% and 82% (X-Gal staining, not shown), in good agreement with previous observations using this class of vectors [16].

### Effects of rAAV-FLAG-h*SOX9 *treatment on the proliferation and viability of undifferentiated hMSCs

The candidate rAAV-FLAG-h*SOX9 *vector was next applied over time to undifferentiated monolayer cultures of hMSCs to investigate potential undesirable effects of the gene-transfer method and of the candidate factor on cell proliferation and viability compared with control conditions (rAAV-*lacZ *transduction and absence of vector treatment). Application of rAAV-FLAG-h*SOX9 *did not significantly modify the cell numbers, viability, proliferation rates (WST-1 assay), or DNA contents of the cultures compared with control treatments (*P *≥ 0.106) (Table 1). Over time, these parameters decreased in all the conditions tested (*P *≤ 0.029), as previously observed by using similar controls in experimental settings [16].

**Table 1:** Analyses in undifferentiated monolayer cultures of human mesenchymal stem cells (hMSCs)

Assay	No vector		rAAV-*lacZ*		rAAV-FLAG-h*SOX9*	
	
	Day 7	Day 21	Day 7	Day 21	Day 7	Day 21
Viable cells	1,225 (50)	855 (25)^a^	1,250 (45)	835 (20)^a^	1,220 (40)	840(25)^a^
Viability (%)	92 (2)	53 (2)^a^	91 (3)	51 (2)^a^	93 (3)	53 (2)^a^
WST -1 (OD^450 nm^)	0.34 (0.02)	0.27 (0.02)^a^	0.32 (0.03)	0.24 (0.01)^a^	0.35 (0.03)	0.26 (0.02)^a^
DNA (ng/mg total proteins)	0.19 (0.02)	0.11 (0.01)^a^	0.21 (0.02)	0.12 (0.01)^a^	0.18 (0.01)	0.14 (0.01)^a^

Values are given as mean (SD) by using a vector dose of 40 μl. ^a^Statistically significant compared with earlier time point.

### Efficient and sustained *SOX9 *overexpression in chondrogenically differentiated cultures of hMSCs via rAAV

hMSCs were next transduced in chondrogenically differentiated (aggregate) cultures over time with rAAV-FLAG-h*SOX9 *compared with control treatments (rAAV-*lacZ *or absence of vector application) to determine the ability of rAAV to mediate *SOX9 *overexpression in a three-dimensional environment adequate for chondrogenic differentiation. In good agreement with the findings in monolayer culture, prolonged FLAG tag expression was seen only in aggregates treated with rAAV-FLAG-h*SOX9 *after 5 days and for up to 21 days, again reaching high transduction efficiencies (80% to 85%) [16], whereas SOX9 immunoreactivity was noted in all types of aggregates as a result of the chondrogenic induction [50], although specific staining was more intense (about ninefold) when the *SOX9 *vector was provided (Figure 1b). No apparent difference in SOX9 immunostaining was noted between control conditions, suggesting that gene transfer via rAAV did not alter the potency of hMSCs, consistent with previous observations with this class of vector [8,16,34].

### Effects of rAAV-FLAG-h*SOX9 *treatment on the proliferative, metabolic, and chondrogenic properties of induced hMSCs

rAAV-FLAG-h*SOX9 *was then used to evaluate the effects of the transcription factor via rAAV administration on the proliferative, biosynthetic, and differentiative activities of hMSCs in conditions of chondrogenic induction (aggregate cultures) over time compared with control conditions (rAAV-*lacZ *transduction and absence of vector treatment).

**Figure 2:**
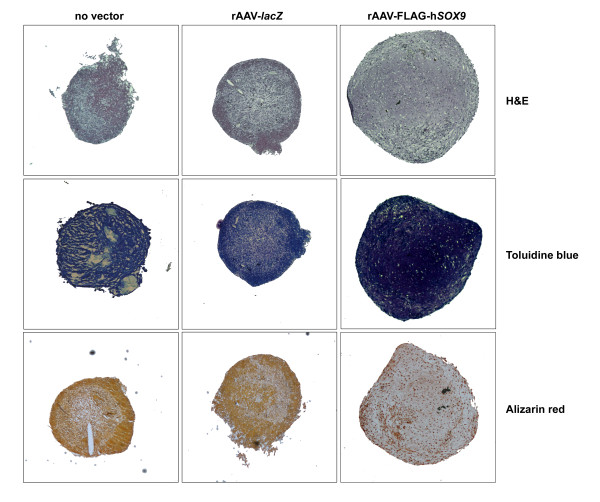
**Histologic analyses in chondrogenically differentiated aggregate cultures of human mesenchymal stem cells (hMSCs)**. Aggregate cultures were prepared and transduced with rAAV-*lacZ *or rAAV-FLAG-h*SOX9*, as described in Figure 1, or left untreated, and processed on day 21 for histologic staining with H&E, toluidine blue, and alizarin red, as described in Materials and methods. All at magnification ×4.

Application of rAAV-FLAG-h*SOX9 *significantly increased the diameters of the aggregates over time and compared with control conditions (up to 1.6-fold; always *P *≤ 0.001) (Figure 2 and Table 2), in which instead significant decreases were noted during the course of the evaluation (up to 1.03-fold; always *P *≤ 0.001) without difference at similar time points (*P *≥ 0.125), as previously reported [16]. Interestingly, this effect of *SOX9 *treatment was not accompanied by increases in the cell densities (H&E staining), proliferation rates (WST-1 assay), or DNA contents in the aggregates (Figure 2 and Table 2), as no difference was found between *SOX9*-, rAAV-*lacZ*-treated, and untreated aggregates at similar time points for these parameters (*P *≥ 0.389) that decreased over time in all types of aggregates (up to 2.2-fold; *P *≤ 0.002), consistent with the findings in monolayer culture and with previous observations [16].

**Table 2:** Analyses in chondrogenically differentiated aggregate cultures of human mesenchymal stem cells (hMSCs)

Assay	No vector		rAAV-*lacZ*		rAAV-FLAG-h*SOX9*	
	
	Day 7	Day 21	Day 7	Day 21	Day 7	Day 21
Diameter (μm)	632 (6)	618 (4)^c^	630 (2)	614 (3)^c^	729 (4)^a, b^	982 (5)^a, b, c^
Cell density (cells/mm^2^)	433 (7)	424 (2)^c^	438 (4)	426 (1)^c^	437 (4)	425 (5)^c^
WST-1 (OD^450 nm^)	1.33 (0.17)	0.62 (0.12)^c^	1.29 (0.21)	0.67 (0.12)^c^	1.31 (0.15)	0.65 (0.14)^c^
DNA (ng/mg total proteins)	0.92 (0.02)	0.87 (0.02)^c^	0.90 (0.01)	0.86 (0.01)^c^	0.92 (0.01)	0.88 (0.02)^c^
Toluidine blue intensity (pixels)	ND	167 (4)	ND	169 (5)	ND	914 (7)^a, b^
Proteoglycans (ng/mg total proteins)	0.61 (0.02)	0.79 (0.04)^c^	0.59 (0.03)	0.80 (0.02)^c^	3.37 (0.06)^a, b^	4.68 (0.07)^a, b, c^
Proteoglycans/DNA (ng/ng)	0.67 (0.04)	0.91 (0.03)^c^	0.66 (0.03)	0.93 (0.02)^c^	3.67 (0.04)^a, b^	5.32 (0.08)^a, b, c^
Type II collagen intensity (%)	ND	51 (3)	ND	49 (2)	ND	85 (3)^a, b^
Type II collagen (pg/mg total proteins)	13 (2)	18 (3)^c^	14 (2)	19 (3)^c^	75 (4)^a, b^	112 (3)^a, b, c^
Type II collagen/DNA (pg/ng)	14 (2)	21 (2)^c^	15 (1)	22 (3)^c^	81 (4)^a, b^	127 (8)^a, b, c^
Type I collagen intensity (%)	ND	44 (2)	ND	42 (4)	ND	6 (2)^a, b^
Type X collagen intensity (%)	ND	45 (3)	ND	46 (2)	ND	8 (2)^a, b^
Alizarin red intensity (pixels)	ND	57 (3)	ND	56 (2)	ND	5 (2)^a, b^
ALP activity (pg/mg total proteins)	ND	57 (2)	ND	55 (1)	ND	25 (1)^a, b^
ALP/DNA (pg/ng)	ND	66 (2)	ND	64 (3)	ND	28 (1)^a, b^

Values are given as mean (SD) with a vector dose of 100 μl. ALP, alkaline phosphatase; ND, not done. Statistically significant compared with ^a^condition without vector treatment, ^b^rAAV-*lacZ*, and ^c^earlier time point.

**Figure 3:**
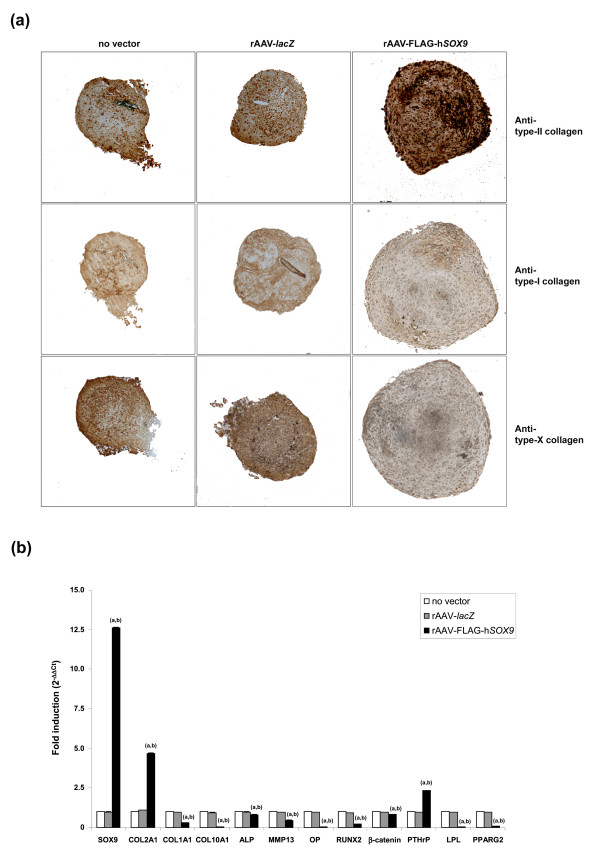
**Expression analyses in differentiated aggregate cultures of human mesenchymal stem cells (hMSCs)**. Aggregate cultures were prepared and transduced with rAAV-*lacZ *or rAAV-FLAG-h*SOX9*, as described in Figure 1, or left untreated, and processed on day 21 for **(a) **immunodetection of type II, type I, and type X collagen (all at magnification ×4), and **(b) **gene-expression analysis by real-time RT-PCR amplification after total cellular RNA extraction and cDNA synthesis, as described in Materials and methods. The genes analyzed included the transcription factor *SOX9*, and types II, I, and X collagen (COL2A1, COL1A1, COL10A1), alkaline phosphatase (ALP), matrix metalloproteinase 13 (MMP13), osteopontin (OP), the transcription factor RUNX2, β-catenin, parathyroid hormone-related protein (PTHrP), lipoprotein lipase (LPL), and the peroxisome proliferator-activated receptor gamma 2 (PPARG2), with GAPDH serving as a housekeeping gene and internal control (primers are listed in Materials and methods). Ct values were obtained for each target and GAPDH as a control for normalization, and fold inductions (relative to untreated aggregates) were measured by using the 2^-ΔΔCt ^method. Statistically significant compared with (a) condition without vector treatment or (b) rAAV-*lacZ*.

Remarkably, when the aggregates were processed to monitor the differentiative and metabolic activities of hMSCs, successful chondrogenic differentiation was noted in all types of aggregates, as evidenced by toluidine blue staining and type II collagen deposition (Figures 2 and 3a), yet significant increases were noted in the presence of the *SOX9 *vector for the intensities of toluidine blue staining, the proteoglycan contents (before and after normalization to the DNA contents), and the intensities of type II collagen immunostaining and contents (before and after normalization) compared with control conditions (Figures 2 and 3a and Table 2) (up to 6.2-fold increase; always *P *≤ 0.001). Notably, the proteoglycan and type II collagen contents significantly increased over time in all types of aggregates (up to 1.6-fold; always *P *≤ 0.001). Also interestingly, no difference was seen between the rAAV-*lacZ*-treated and untreated aggregates for these parameters at similar time points (*P *≥ 0.439) [16]. In marked contrast, application of the *SOX9 *vector compared with control conditions caused a significant reduction in the intensities of immunostaining for type I and type X collagen, in those of alizarin red staining (up to 9.5-fold; always *P *≤ 0.001), and in the ALP activities (before and after normalization) (up to 2.4-fold; always *P *≤ 0.001) (Figures 2 and 3a and Table 2). Also interestingly, no difference occurred between rAAV-*lacZ*-treated and untreated aggregates for any of these parameters when comparing similar time points (*P *≥ 0.570) [16]. In contrast with the findings for type II collagen, the type I and type X collagen contents could not be estimated with ELISA, as the values were always below the levels of detection of the assays.

The findings related to the biochemical, histologic, and immunohistochemical analyses were corroborated by data from a real-time RT-PCR analysis (Figure 3b). Chondrogenic differentiation of hMSCs was observed in all types of aggregates after 21 days, as seen by detection of *SOX9 *and type II collagen expression, yet significant differences were noted between *SOX9 *and control conditions (rAAV-*lacZ *or no vector treatment) (up to 13-fold higher *SOX9 *expression levels and up to 4.7-fold higher COL2A1 expression levels in the presence of rAAV-FLAG-h*SOX9*; always *P *≤ 0.001). Real-time RT-PCR analysis also confirmed the decreased profiles of type I, type X collagen, and ALP when overexpressing *SOX9 *compared with control treatments (up to 3.3-, 2.5-, and 1.3-fold, respectively; always *P *≤ 0.001). Strikingly, the analysis further revealed opposing effects of *SOX9 *treatment on the expression of MMP13 (up to 2.2-fold), OP (up to 33.3-fold), RUNX2 (up to 4.8-fold), β-catenin (up to 1.2-fold), LPL (up to 33.3-fold), and PPARG2 (up to 14.3-fold) (always *P *≤ 0.001), while showing activating effects on PTHrP (up to 2.4-fold; *P *≤ 0.001) compared with control conditions. Again as previously reported, no difference was observed between rAAV-*lacZ*-treated and untreated aggregates for any of the markers analyzed here (*P *≥ 0.417) [16].

### Effects of rAAV-FLAG-h*SOX9 *treatment on the osteogenic differentiation potential of hMSCs

The candidate *SOX9 *vector was next provided to osteogenically differentiated hMSCs over time to estimate further the effects of the transcription factor via rAAV application on the potential for osteogenic differentiation of the cells compared with control conditions (rAAV-*lacZ *transduction and absence of vector treatment).

**Figure 4:**
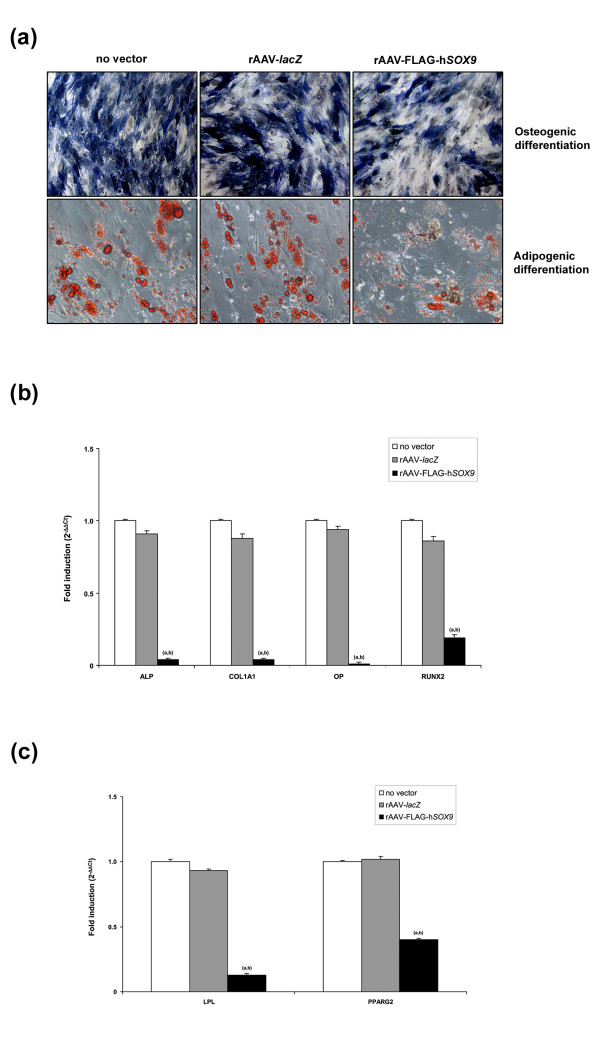
**Analyses in osteogenically and adipogenically differentiated cultures of human mesenchymal stem cells (hMSCs)**. Cells in monolayer cultures were transduced with rAAV-*lacZ *or rAAV-FLAG-h*SOX9 *(100 μl each vector) or left untreated and induced toward osteogenic or adipogenic differentiation, as described in Materials and methods. Cultures were processed on day 21 for **(a) **ALP staining (osteogenesis; magnification ×4) and Oil Red O staining (adipogenesis; magnification ×10) and **(b) **and **(c) **gene-expression analysis with real-time RT-PCR amplification, as described in Figure 3. The genes analyzed included *ALP, COL1A1, OP*, and *RUNX2 *for osteogenically differentiated cultures (b) and *LPL *and *PPARG2 *for adipogenically differentiated cultures (c), with GAPDH serving as a housekeeping gene and internal control in both cases. Ct values were obtained for each target and GAPDH as a control for normalization, and fold inductions (relative to untreated cultures) were measured by using the 2^-ΔΔCt ^method. Statistically significant compared with (a) condition without vector treatment or (b) rAAV-*lacZ*.

Successful differentiation was noted in all types of induced cultures, as evidenced by ALP staining (Figure 4a). Nevertheless, application of rAAV-FLAG-h*SOX9 *significantly decreased the percentage of stained areas after 21 days compared with control conditions (42% ± 2% versus 83% ± 2% or 84% ± 3% with rAAV-*lacZ *or without vector, respectively; that is, an up to twofold decrease; always *P *≤ 0.001). This observation was corroborated when estimating the ALP activities of the cultures (5.8 ± 0.4 nmol/mg total proteins with rAAV-FLAG-h*SOX9 *versus 42.4 ± 2.3 or 39.2 ± 1.6 nmol/mg total proteins with rAAV-*lacZ *or without vector (that is, an up to 7.3-fold decrease; always *P *≤ 0.001) and with real-time RT-PCR analysis (up to 25-fold decrease in ALP expression; *P *≤ 0.001) (Figure 4b). Treatment with *SOX9 *also decreased the expression levels of type I collagen, OP, and RUNX2 compared with control transductions (up to 100-fold; *P *≤ 0.001) (Figure 4b). Once again, no difference was noted between rAAV-*lacZ*-treated and untreated cultures for any of the markers analyzed here (*P *≥ 0.570).

### Effects of rAAV-FLAG-h*SOX9 *treatment on the adipogenic differentiation potential of hMSCs

Finally, the candidate *SOX9 *vector was provided to adipogenically differentiated hMSCs over time to validate further the effects of the transcription factor through rAAV application on the potential for adipogenic differentiation of the cells compared with control conditions (rAAV-*lacZ *transduction and absence of vector treatment).

Successful differentiation was achieved in all types of induced cultures, as seen by the accumulation of lipid droplets after staining with Oil Red O (Figure 4a). However, *SOX9 *gene transfer significantly decreased the percentage of stained areas after 21 days compared with control conditions (36% ± 2% versus 53% ± 2% or 51% ± 3%, that is, an up to 1.5-fold decrease; always *P *≤ 0.001). This finding was substantiated by real-time PCR expression analysis of LPL and PPARG2, showing significantly decreased levels in the presence of rAAV-FLAG-h*SOX9 *compared with control treatments (up to 7.7-fold; *P *≤ 0.001) (Figure 4c). Again, no difference was noted between rAAV-*lacZ*-treated and untreated cultures for any of the markers tested (*P *≥ 0.389).

## Discussion

Transplantation of progenitor cells like mesenchymal stem cells (MSCs) from the bone marrow, with an innate potential for chondrogenic differentiation, is a promising strategy to treat articular cartilage defects in patients [3]. Yet, the use of MSCs in such settings is still restrained by the low percentage of cells that enter appropriate chondrocyte lineage-differentiation pathways to produce a reparative tissue of proper quality. It is well known that MSC-derived cells tend to undergo premature terminal differentiation, hypertrophy, and ossification [4,5]. Such limitations might be overcome by directing the cells toward an adequate phenotype by application and stable expression of candidate genes capable of controlling chondrocyte differentiation. Among the potentially beneficial agents, the transcription factor SOX9 is a strong candidate to adjust chondrogenesis, as a key regulator of chondrocyte differentiation and cartilage formation [22] that can delay hypertrophic maturation at certain stages of differentiation [23-27]. Instead of using classic nonviral [26,30], adeno-, retro-, and lentiviral vectors [24,31-33], we focused on rAAV systems that advantageously genetically modify hMSCs at very high efficiencies and for extended periods without affecting their potential for differentiation [8,16,34]. This finding was confirmed here when applying the rAAV-FLAG-h*SOX9 *vector to undifferentiated monolayer and chondrogenically differentiated hMSC cultures (70% to 85% transduction efficiencies for up to 21 days, with about a ninefold difference in *SOX9 *expression levels compared with control treatments that showed a similar evolution for all parameters in the evaluation). Equally important, we further demonstrate that the efficient and sustained *SOX9 *expression levels achieved here with rAAV were capable of promoting and enhancing chondrogenic differentiation of hMSCs in suitable aggregate cultures, with an increased production of major extracellular matrix components (proteoglycans, type II collagen) compared with control conditions, as already seen in human osteoarthritic chondrocytes [43] and in agreement with the properties of this factor [22,24,26,27,31,32]. Interestingly, administration of rAAV *SOX9 *did not further modify the levels of proliferation and viability of hMSCs in all the systems tested compared with control treatments, as also reported with chondrocytes [43], and instead, these parameters decreased over the course of the evaluation. It remains to be seen whether too elevated levels of *SOX9 *expression will not cause toxicity on cells transduced through rAAV [51]. This is, however, consistent with previous observations when expanding similar controls of hMSC transduction [16] and, more important, with findings showing the lack or opposing effects of *SOX9 *on the proliferation and cell-cycle progression of hMSCs in the adult [23,31,32].

Strikingly, the present results also indicate that prolonged, elevated rAAV-mediated expression of *SOX9 *significantly reduced the expression and activities of several markers of hypertrophy and terminal or osteogenic differentiation (type I and type X collagen, ALP, MMP13, OP, matrix mineralization), concordant with previous reports showing contrasting effects of SOX9 on osteogenesis, bone formation, terminal differentiation, and calcification and on the expression of these markers [23-27,31,52-58]. Remarkably, these effects of *SOX9 *treatment via rAAV were associated with significant decreases in the levels of RUNX2, a transcription factor essential for bone formation, terminal maturation, and mineralization that stimulates the expression of osteoblast-related genes (*COL1A1, COL10A1, ALP, MMP13, OP*) [53,55,56,58-62], again in good agreement with the effects of SOX9 on RUNX2 expression [31,63,64]. Interestingly, Ikeda *et al. *[24] reported that *SOX9 *gene transfer in hMSCs failed to suppress the expression of such hypertrophic and osteogenic markers. However, it is important to mention that, in this previous study, less efficient adenoviral vectors were used and mediated gene expression for about 5 days, whereas sustained and very high levels of transgene *SOX9 *expression were detected here for at least 21 days. Of further note, we also observed that application of rAAV-FLAG-h*SOX9 *led to a decrease in β-catenin expression, a mediator of the Wnt signaling pathway known to stimulate osteoblast lineage differentiation [65]. In addition, we noted that the vector increased the levels of PTHrP, an inhibitor of hypertrophic maturation and calcification that has a significant impact on the regulation of gene expression for COL1A1, COL10A1, ALP, and RUNX2, delays bone formation [15,53,55,56,58,66-72], and activates SOX9 transcriptional activities [67]. The effects of *SOX9 *evidenced here on these signaling pathways are consistent with reports showing enhanced β-catenin degradation and PTHrP activation mediated by the cartilage-specific transcription factor [23,52,73].

Altogether, the data demonstrate that concurrent activation and inhibition of different signaling pathways by rAAV *SOX9 *gene transfer might permit a significant reduction of osteogenic processes in hMSCs. Still, in the present study, evaluations were not performed beyond day 21, and it remains to be seen whether the *SOX9*-transduced cells will not undergo hypertrophy and terminal or osteogenic differentiation over time if they lose *SOX9 *expression [28,29], an issue that might have consequences for an adequate treatment of cartilage lesions. Also noteworthy, the candidate treatment here also significantly decreased the expression of adipogenic markers (accumulation of lipid droplets, LPL, and PPARG2 levels), allowing containing the adipogenic differentiation of hMSCs, again in good agreement with previous findings [27,57]. To our best knowledge, this is the first evidence showing that overexpression of *SOX9 *via rAAV stimulates hMSC chondrogenic differentiation with an important delay in terminal differentiation and hypertrophy, while affecting osteogenic and adipogenic differentiation over a continuous period. Apart from SOX9, the use of other members of the SOX family (SOX5, SOX6) [52,56,74] might be of further benefit to favor chondrogenic versus osteo-/adipogenic differentiation of hMSCs, as proposed by various groups who delivered the SOX trio by more classic, less efficient nonviral or adenoviral vectors [24,30]. Delivery of additional factors displaying proliferative activities might be also valuable to generate high numbers of hMSCs for transplantation in articular cartilage defects. Among various agents with such effects, IGF-I [14], FGF-2 [16,17], hTERT [19,20], or Bcl-xL [21] may be potentially provided along with *SOX *sequences. Again, rAAV are powerful vectors, as they conveniently permit separate expression of multiple genes at the same time within their targets [42].

In addition, it will be important to test further whether transplantation of rAAV *SOX9*-modified MSCs in articular cartilage defects allows for an effective healing of the lesions *in vivo*, in association with competent chondrogenic differentiation that avoids premature terminal differentiation as noted *in vitro*. Interesting findings have been reported by Cao *et al. *[31], who showed that implantation of MSCs modified to overexpress *SOX9 *in a polyglycolic acid (PGA) scaffold led to better repair of osteochondral defects in rabbits, although the gene-transfer system used was a relatively low efficiency adenoviral vector compared with rAAV that might prove even more effective because of higher levels and duration of transgene expression. Regarding the value of the present approach for cartilage repair, this strategy with rAAV will have to be translated in rabbit MSCs before transplantation of genetically modified cell platforms within cartilage defects *in vivo*. Parameters to consider will include the amounts of cells to provide, the potential use of control elements to contain transgene expression (lineage-specific or regulatable promoters), and the selection of the best-suited supportive matrix for cell containment in the lesions. Also, long-term evaluations will be necessary to test the mechanical quality of the repair tissue within the defects, as other cells (periosteum-, perichondrium-, adipose-, muscle-derived stem cells, bone marrow aspirates, tissue grafts, or even chondrocytes) might be applied as engineered platforms [75-78] compared with direct gene-transfer strategies [35,79,80].

## Conclusions

The results of the present study indicate that gene transfer through therapeutic rAAV might be largely beneficial to produce an MSC-derived cell population with a strong potential for proper chondrogenic differentiation, as a means to develop indirect gene- and cell-based approaches to treat articular cartilage defects *in vivo*.

## Abbreviations

ALP: alkaline phosphatase; COL2A1: type II collagen; LPL: lipoprotein lipase; MMP13: matrix metalloproteinase 13; MSCs: mesenchymal stem cells; OP: osteopontin; PPARG2: peroxisome proliferator-activated receptor γ2; PTHrP: parathyroid hormone-related protein; rAAV: recombinant adenoassociated virus; RUNX2: runt-related transcription factor 2; SOX9: sex-determining region Y-type high-mobility group box 9.

## Competing interests

The authors declare that they have no competing interests.

## Authors' contributions

JKV, ME, HM, GS, and MC performed the experiments and collected the data. JKV, ME, HM, DK, and MC analyzed the data. MC designed the study and drafted and edited the manuscript. All authors approved the final manuscript.
